# Cognitive training in older adults with Mild Cognitive Impairment: Impact on cognitive and functional performance

**DOI:** 10.1590/S1980-57642009DN30200010

**Published:** 2009

**Authors:** Paula Schimidt Brum, Orestes Vicente Forlenza, Mônica Sanches Yassuda

**Affiliations:** 1 Bachelor in Gerontology EACH-USP, São Paulo, SP, Brazil.; 2 MD, Psychiatrist. Collaborating Professor and PhD in Medicine from the Department of Psychiatry of the Faculty of Medicine of the University of São Paulo, São Paulo, SP, Brazil.; 3 PhD, Assistant Professor of the Gerontology EACH-USP. Researcher at the Psychogeriatric Outpatient Unit, LIM-27, IPq FMUSP, São Paulo, SP, Brazil.

**Keywords:** Mild Cognitive Impairment, cognitive training, aging, cognition, functionality

## Abstract

Aging is associated with cognitive decline, yet this does not prevent older adults from finding ways to compensate for age-related deficits. Earlier studies have shown that cognitively unimpaired older adults can benefit from training programs. The efficacy of cognitive interventions among older adults without dementia but with cognitive decline (mild cognitive impairment, MCI) has not yet been widely tested.

**Objectives:**

To evaluate the impact of 8-session cognitive training on the cognitive and functional performance of older adults with MCI.

**Methods:**

16 older adults diagnosed with MCI received cognitive training (18 participated as controls). All participants were assessed pre and post intervention using the Short Cognitive Test (SKT), Direct Assessment of Functional Scale Revised (DAFS-R), Geriatric Depression Scale (GDS), and Clock Drawing Test (CDT).

**Results:**

A significant improvement was observed in the study group between pre and post-test in attention (SKT), time orientation, shopping skills and dealing with finances (DAFS-R) along with reduced depressive symptoms (GDS).

**Conclusion:**

These results indicate the importance of non-pharmacological interventions for older adults with MCI to help compensate for cognitive decline.

## Abstract (pt)

O envelhecimento está associado ao declínio de algumas funções cognitivas, entretanto, este fato não impede que a pessoa idosa encontre formas de compensar déficits. Estudos anteriores documentaram que a pessoa idosa sem declínio cognitivo pode beneficiar-se de programas de treino. A eficácia de intervenções cognitivas junto à população idosa sem demência mas com alterações cognitivas (comprometimento cognitivo leve, CCL) ainda não foi testada amplamente.

**Objetivos:**

Avaliar o impacto de treino cognitivo de 8 sessões na funcionalidade e desempenho cognitivo em idosos com CCL.

**Métodos:**

16 idosos com CCL receberam treino cognitivo e 18 participaram como grupo controle; pacientes e controles foram avaliados antes e depois da intervenção com o Short Cognitive Test (SKT), Direct Assessment of Funcional Scale Revised (DAFS-R), Escala de Depressão Geriátrica (GDS) e o Teste do Desenho do Relógio (TDR).

**Resultados:**

Foi observada melhora significante no grupo experimental do pré para o pós-teste no desempenho em atenção (SKT), orientação temporal, habilidade para fazer compras e lidar com dinheiro (DAFS-R) e diminuição nos sintomas depressivos (GDS).

**Conclusões:**

Estes resultados destacam a importância da intervenção não-farmacológica em idosos com CCL para compensar déficits cognitivos.

Amid the global phenomenon of aging, the volume of research dedicated to investigating the peculiarities of old age has risen significantly.^1^ At the same time, there has been growing interest in cognitive aging. For decades, scientific studies have described frequent changes in cognition of the elderly. Most common of these changes affect episodic memory and executive functions.^2,3^

Researchers now recognize 4,5 that older adults may present a range of different cognitive profiles, such as:

1) stability in activities of daily living (ADLs) and on neuropsychological tests;
2) preserved activities of daily living (ADLs) but performance deficit on cognitive tests; or even,
3) compromised ADLs and performance deficit on cognitive tests. The first profile indicates senescence, the second Mild Cognitive Impairment (MCI), and the third dementias.

MCI is considered by many scholars as an intermediate stage between normal and pathologic cognitive aging.^4-6^

It is known that older adults diagnosed with MCI present a higher risk of developing dementias than healthy elderly.^4,5,7^ Studies have shown pharmacologic intervention with cholinesterase inhibitors to be largely ineffective in delaying progression to dementias.^8,9^

The MCI concept has led to debate regarding the value of non-pharmacologic interventions in pre-clinical stages of dementias. The impact of cognitive training in elderly with MCI has yet to be elucidated. It has been proposed that this group should benefit most from cognitive interventions because this population presents greater cognitive decline than healthy elderly and therefore should show a more marked improvement in pre and post intervention scores upon returning to normal cognitive levels. On the other hand, a lesser degree of plasticity or gain following intervention may be expected since this group of elderly can present with a pathologic process characterized by neuronal death.

According to a review carried out by Belleville,^10^ cognitive training appeared to optimize cognitive functions in elderly diagnosed with MCI. The results of the review showed that training in elderly with MCI had greater effect in the younger old and more cognitively preserved individuals, and it may have the potential to slow the rate of cognitive and functional decline. Belleville^10^ also highlighted the need for further controlled studies on the topic and the need to toward devise effective intervention protocols and efficacy measures for these interventions.

In a Brazilian review article, Miotto and colleagues^11^ reported that studies available in the literature on MCI training showed significant improvement in cognitive test performance after non-pharmacologic intervention in this population. Olchik^12^ conducted the first Brazilian study on cognitive training in MCI involving 112 elderly. Although the cognitive enhancement following training proved modest, the outcomes showed that, as a group, performance was restored to levels comparable to those of preserved elderly. Olchik^12^ stated that persons with MCI exhibited neural plasticity, making cognitive training a viable form of non-pharmacologic treatment.

Controlled studies on training should be performed in older adults with cognitive deficits to further investigate neural plasticity in this population.^10^ The effects of training on ADLs among MCI patients remain unknown.

The aim of the present study was to assess the effects of cognitive training in older adults diagnosed with MCI. Cognitive and functional variables were used as a measure of intervention efficacy.

## Methods

### Materials and methods

This study was part of a larger project conducted on the neuroprotective effects of lithium. In the main research, older adults diagnosed with MCI by a multi-disciplinary team were randomized to receive lithium or placebo for a one-year period. In parallel with the main study period, all participants followed the non-pharmacologic intervention program described in this study. Thirty-four participants from the Lithium study who had completed 12 months of follow up by September 2008, were invited to take part in this intervention and were randomly assigned to a study group (SG, i.e. cognitive training) or a control group (CG). The researchers involved in the training study were blinded to the assigned group of the participants in the lithium study protocol until the end of the data collection phase.

The participants of both studies were recruited from the Psychogeriatrics Outpatient Unit of LIM-27, IPq, FMUSP, where they were assessed by a multi-disciplinary team comprising psychiatrists, neuropsychologists, geriatricians, and diagnoses were reached through consensus discussions. DSM-IV criteria were used to rate dementias, while the criteria of Petersen and colleagues^13^ were employed for MCI. The elderly visiting this outpatient unit regularly undergo a vast battery of psychometric instruments which assess cognitive and functional skills. The test battery applied includes the Cambridge Examination for Mental Disorders of the Elderly (CAMCOG)^14^ and the Informant Questionnaire on Cognitive Decline in the Elderly (IQCODE),^15^ Rivermead Behavioral Memory Test (RBMT),^16^ Fuld Object–Memory Evaluation (FOME)^,^^17^ Short Cognitive Test (SKT),^18,19^ Nelson’s Modified Cards Sorting Test,^20^ Trail-making tests A and B^21^ and the Vocabulary and Block design subtests of the WAIS-R battery.^22^

The participants were also submitted to laboratory exams in order to detect any treatable causes of cognitive decline. Neuroimaging exams were performed when the team deemed necessary.

In terms of medications used by the study participants, both groups were on similar drug regimens. Many subjects were taking medications for hypertension (10 in the CG and 12 in the SG), and others for diabetes (2 in the CG and 4 in the SG). Seven individuals in the CG and 4 in the SG were taking anti-depressants. Three participants in the CG and 4 in the SG were also in use of cholinesterase inhibitors. The use of other drugs was reported less frequently: for dyslipidemia (3 in CG and 4 SG), osteoporosis (1 in CG and 1 SG), rheumatism (1 in CG and 1 SG), arthrosis (1 SG), gastritis (3 SG), and insomnia (3 SG).

### Materials

The following instruments were used to assess the efficacy of the cognitive treatment given in this study: the DAFS-R functional assessment battery, Direct Assessment of Functional Status,^23,24^ the SKT (Short Cognitive Test)^18,19^ the 15-item Geriatric Depression Scale,^25^ the Clock Drawing Test.^26,27^

The DAFS-R is a functional performance measure developed to assess functionality in older adults and indicate their capacity to perform activities of daily living. It is made up of several sub-tests which elicit the elderly subject to perform tasks such as making a telephone call, simulated supermarket shopping, filling out a check, etc. The value of this battery is that it does not depend on reports from the interviewee or family members regarding the patient’s functional status but instead relies on empirical performance observed on simulated tasks.^23^

This instrument has been adapted to Portuguese,^24^ and is in the process of validation and reliability testing. The following domains of the DAFS-R were applied in the current study: Time orientation (0 to 16), Communication (0 to 15), Dealing with finances (0 to 32), Shopping skills (0 to 20), yielding a total possible score of 83 points. The higher the score on the DAFS-R, the better the functional performance of the individual. Getting dressed and Feeding were not included in this study because ceiling effect was observed for these domains in the study by Pereira and colleagues,^24^ and they proved unable to differentiate between the three diagnostic groups (controls, MCI, and Alzheimer’s disease).

The SKT is commonly used to identify and monitor cognitive decline. The test consists of 9 sub-items, 3 of which are related to memory and 6 to attention. Scores on the attention domain range from 0 to 18 points and on the memory domain from 0 to 9 points. The total SKT score therefore ranges from 0 to 27 points with lower scores being suggestive of cognitive preservation. There are 5 different versions of this scale and versions A and E were used in this study (in a bid to avoid the retest effect). The test lasts 15 minutes on average and the participant’s age and intelligence-score (estimated IQ) is needed in order to carry out the analysis. The latter data was obtained from the data base of the Psychogeriatrics Outpatient Unit. The SKT has been previously validated for use in the Brazilian aged population.^19^

The short version of the Geriatric Depression Scale (GDS) (containing 15 questions) was applied in order to identify participants with depression and exclude them from the analysis, and also to verify whether training exerted any effect on the mood of participants. A cut-off of six points or more was adopted as being suggestive of depression.^25^

The Clock Drawing Test was used (CDT) to assess the visuo-constructional capacity of participants. This test entails asking older adults to draw a clock face and mark 10 minutes past eleven. The criteria by Sunderland and colleagues^28^ were used to score the test on a scale of 0 to 10 points.

### Procedures

All participants signed a free and informed consent term previously approved by the institution’s ethics committee overseeing the study, on entry to the main study on the neuroprotective effect of lithium. The cognitive intervention study was a sub-project of this main research.

The training protocol applied to SG patients involved an educational component on memory and aging, and a practical component on cognitive tasks. The aim of the protocol was to implement the compensatory strategy (categorization) for episodic memory tasks through ecologic activities which simulated activities of daily living. The training also included tasks involving executive functions by means of simulated tasks of giving and cheking change. Each training session comprised the following elements:

1. Orientation in time and space – performed using external aids such as a calendar and the day’s newspaper, whereby participants determined the current day, month and year;
2. Presentation of the names of participants and the researcher. Verbal associations were elicited between the person’s name and their appearance.
3. Visual and auditory attention exercises. The visual attention task entailed identifying details in photographs, letters or figures amongst several stimuli spread out on a single printed sheet, as well as spotting differences between two similar photographs, among other activities. The auditory attention exercises included tasks such as detecting words in songs, and identifying whether two consecutively repeated sequences of numbers or words matched or differed;
4. Memory exercises using visual aids – the categorization strategy with ecological tasks was used, i.e. simulated activities of daily living, graded from simple to more complex. Grocery items were used in the early stages of training progressing to supermarket item lists only, by the end of training.
5. Transfer task – practical tasks from activities of daily living were used, such as simulating a to the supermarket, giving and checking change, etc. The older adults were expected to calculate the total cost of the purchase and offer change or check the change received.

The training sessions in the Study Group (16 participants) lasted two hours each, and were run twice weekly over a one-month period, in a total of 8 sessions. For ethical reasons, the control group (18 participants) was given 4 training sessions at study endpoint.

### Data analysis

In order to ascertain whether the variables had a normal distribution, Kolmogorov Smirnov tests were performed. The majority of variables presented a normal distribution except for Memory on the SKT, and Time Orientation and Communication on the DAFS-R tests. Parametric tests were applied first and analyses were then repeated using non-parametric tests for the above-mentioned variables. Student’s t test for independent samples was employed to compare the two groups at pre-test. This test was also used to compare the deltas (post-test minus pre-test performance) between the two groups for the variables analyzed. Student’s t test for paired samples was used to compare pre and post test performance for each group separately. The data were analyzed using the SPSS statistical programme for Windows version 9.0, and the level of significance adopted for the statistical tests was 5%.

## Results

The gender distribution for the 34 participants was 25 women versus 9 men. The study group comprised 16 subjects, 2 men and 14 women, while the control group contained 18 subjects, 7 men and 11 women.

The study group (SG) denotes the group of older adults who were submitted to initial assessment, received the intervention, i.e. took part in the 8 cognitive training sessions, and then underwent reassessment. The control group (CG) refers to participants who underwent initial assessment, did not take part in the training sessions, and were then reassessed. For ethical reasons, the CG received cognitive training after the final reassessment.

Regarding socio-demographic data, Table 1 shows means, standard deviations, and p-value for the variables age and schooling, the Geriatric Depression Scale and SKT in both SG and CG on the pre-test. These data showed groups were similar at baseline for age, schooling, depressive symptoms and cognitive performance.

**Table 1:** Means and standard deviations (in parenthesis) for socio-demographic and cognitive data.

	Study group n=16	Control group n=18	p value
Age	73.3 (5.8)	74 (5.1)	0.58
Schooling	9.4 (6.0)	9.5 (5.1)	0.64
GDS	2.8 (2.0)	2.6 (2.4)	0.26
SKT total	4.7 (2.8)	4.1 (2.4)	0.33

p value refers to Student's t test for independent samples.

This study sought to determine which MCI subtypes were present in the sample and to identify any differences in distribution of these subtypes between the SG and CG. Table 2 shows the distribution of MCI subtype diagnosed for participants in the CG and SG. The results show that participants were evenly distributed in terms of MCI subtypes, where amnestic MCI was the most frequent for both groups.

**Table 2:** Number of participants by MCI type and group - raw data and percentage (in parenthesis).

	Amnestic MCI	Multiple Domain MCI with no memory deficit	Amnestic Multiple Domain MCI
Study group	7 (43.7%)	3 (18.7%)	6 (37.5%)
Control group	6 (33.3%)	7 (38.8%)	5 (27.7%)

Study group (SG) n=16, Control group (CG) n=18.

Out of the 34 older adults who took part in this study, 9 were receiving Lithium. Of this nine, 7 were randomly assigned to the CG and 2 to the SG. Table 3 shows the number of participants who received lithium in the CG and SG together with those who did not receive the medication. The effect of lithium on cognition in this study was assessed using two methods. First, pre-test performance of participants who took lithium (n=9) was compared to those who had not taken lithium (n=25), revealing no significant differences in cognitive and functional performance or mood between the two groups. Second, the deltas of these variables were compared for participants who took lithium (n=2) against the deltas of those who did not (n=14) in the SG (trained group). No significant differences from pre to post test performance for the variables were found. Based on these results, the lithium versus no lithium condition was excluded from subsequent analyses.

**Table 3:** Description of participants treated with lithium.

	Lithium received	Lithium not received
Study group	2	14
Control group	7	11

Study group n=16 and Control group n=18.

Table 4 depicts the means and respective standard deviations of scores on the SKT, DAFS-R, CDT and GDS instruments for the SG and CG on pre and post tests. Statistical significance (p value) refers to Student’s t test for paired samples, which compared pre and post test performance for each group separately, or to the value obtained from the Wilcoxon’s non-parametric test.

**Table 4:** Means and Standard Deviations (in parenthesis) for the cognitive variables and depressive symptoms at pre and post test.

	Group	Pre-Test	Post-Test	p value
SKT attention	Study group	3.19 (2.46)	2.25 (2.35)	0.03^a^
	Control group	3.44 (2.41)	4.0 (3.48)	0.54^a^
SKT memory	Study group	1.0 (1.6)	1.0 (1.8)	0.71^a^
	Control group	1.3 (1.3)	1.5 (1.8)	0.72^a^
SKT total	Study group	4.2 (2.3)	3.3 (2.6)	0.06^b^
	Control group	4.8 (2.8)	5.5 (3.9)	0.19^b^
DAFS time orientation	Study group	15.12 (1.3)	15.75 (0.68)	0.03^a^
	Control group	15.22 (1.0)	14.78 (1.56)	0.25^a^
DAFS communication	Study group	14.62 (0.88)	14.69 (0.60)	0.71^a^
	Control group	14.28 (1.23)	14.28 (1.23)	0.19^a^
	Study group	23.38 (4.2)	27.75 (3.1)	0.00^a^
DAFS ability to deal with finances	Control group	23.61 (4.84)	22.06 (6.4)	0.09^a^
	Study group	14.19 (2.9)	16.31(2.33)	0.01^a^
DAFS shopping skills	Control group	14.17 (3.6)	14.67(3.65)	0.27^a^
	Study group	67.32 (6.48)	74.50 (6.49)	0.00^b^
DAFS total	Control group	67.27 (7.9)	65.00 (9.00)	0.11^b^
	Study group	8.94 (1.61)	9.75 (0.44)	0.04^a^
CDT	Control group	8.28 (1.67)	8.94 (1.21)	0.07^a^
	Study group	2.69 (2.44)	1.81 (2.1)	0.07^a^
GDS	Control group	2.89 (1.97)	3.17 (2.1)	0.57^a^

a Refers to the p value according to the test for paired samples;

b Refers to the p value according to Wilcoxon's non-parametric test.

Although data for both groups appeared similar at baseline assessment, significant changes were observed between initial and final assessments in the SG. Specifically, significant improvements were observed in the SG for attention (SKT), time orientation (DAFS-R), dealing with finances (DAFS-R), shopping skills (DAFS-R) and visuo-construction (CDT). Also in the SG, a reduction in reported depressive symptoms (GDS) and SKT test total was seen which approached statistical significance.

To verify whether groups evolved differently between pre and post test, delta values were calculated for each variable by subtracting the pre-test score from the post test score. The delta value is a measure of gain or benefit from training. Table 5 shows the means and standard deviations for the delta values on the SKT, DAFS-R, CDT and GDS. Analyses of deltas confirmed that the SG had significant improvements compared to the CG on attention (SKT), time orientation and ability to deal with finances (DAFS-R). The improvement in shopping skills, which employ memory, approached a statistically significant level.

**Table 5:** Means and standard deviations (in parenthesis) for the Deltas of the cognitive variables and depressive symptoms.

	Study group	Control group	p value
SKT attention	-0.94 (1.53)	0.55 (2.38)	0.04^b^
SKT memory	-0.06 (0.68)	0.17 (1.39)	0.57^a^
SKT total	-0.88 (1.71)	0.72 (2.23)	0.03^b^
DAFS orientation	0.63 (0.96)	-0.44 (1.62)	0.05^a^
DAFS communication	-0.06 (0.68)	-0.78 (2.49)	0.53^a^
DAFS finances	4.37 (3.21)	-1.56 (3.87)	0.00^a^
DAFS shopping	2.13 (2.95)	0.5 (2.03)	0.06^a^
DAFS total	7.19 (5.3)	-2.28 (5.63)	0.00^b^
GDS	-0.87 (1.82)	0.28 (1.5)	0.12^a^
CDT	0.81 (1.64)	0.67 (1.5)	0.75^a^

SG n=16, CG n =18.;

a Refers to p value according to test for independent samples.;

b Refers to the p value according to the non-parametric Mann-Whitney test.

## Discussion

Several recent studies have shown cognitive training to be an effective form of intervention in older adults with MCI.^29,30^ However, results have been mixed regarding the extent of the impact of such interventions on this population, and their capacity to prevent or delay conversion to dementia.

In the present study, 34 older adults, previously diagnosed with MCI, were invited to take part in an 8-session cognitive intervention. Significant changes were found among SG subjects in time orientation, attention, memory and in some aspects of daily functioning, such as the ability to deal with finances.

Few studies on the effect of cognitive interventions in elderly with MCI are available in the literature. The outcomes of these interventions are controversial, where earlier studies have pointed to efficacy of this mode of intervention^2,30-37^ while others have shown no response or only non-significant mild improvement in performance following intervention programs.^28,30,35^ These discrepancies are largely associated to the different methodologies adopted by each study, both in the approach used to assess intervention efficacy as well as the training protocol employed. The study samples are also heterogeneous among investigations.

The intervention used in this study focused on ecological activities, i.e. activities simulating those of daily living among elderly. Research has highlighted the importance of this type of methodology in assessing the impact of training on participants’ daily lives.^10-12,32,34,36^.

The memory exercises contained in the training protocol were based on concrete objects (product packs aging purchased from the supermarket), used in the shopping simulations. These tasks were repetead several times at increasing levels of difficulty. Results showed no significant changes on the memory subtest of the SKT, which entails memorizing 12 color figures. However, a significant improvement was observed in the shopping subtest of the DAFS-R which involves making a supermarket purchase. Thus, the efficacy of the intervention on similar tasks to those trained was observed, while a lack of transfer to other memory tasks was evident (memorizing figures). Previous studies on training in healthy elderly have also shown that the effects of the interventions tend to be seen in similar tasks to those trained in the intervention.^10,38-41^

Notably, the most marked effects of this intervention were seen in subtests which recruit attention (SKT attention score) and executive functions (DAFS-R - dealing with finances). The training offered numerous opportunities of practice with mental calculations (summing the value of simulated purchases), and checking change, when participants role-played the customer or checkout operator at a supermarket. These findings again suggest intervention effectiveness in cognitive tests which are similar to the activities trained, but also suggest possibly a higher degree of plasticity in executive functions.^12,30,42,43^

These differential findings between memory and attention and executive functions can perhaps be explained by the high number of individuals with amnestic MCI in our sample. It may be harder to change previously compromised functions than preserved functions in elderly with this profile.

Time orientation on the DAFS-R also showed substantial change between pre and post test in the SG. This enhancement may have stemmed from the time orientation tasks performed at the beginning of each session and the activities which involved the use of a clock, in which each participant stated the time displayed, again showing greater effects on similar tasks to the trained ones.^10,39,40^

Changes in pre and post test performance also occurred for the Clock Drawing Test in the SG. These changes may have arisen from the contact with the clock in time orientation tasks, evidencing some degree of transfer between the skills trained (clock use) and those assessed (building a clock). It should be noted that the CDT involves visuo-constructive skills which were not trained in the intervention.^12,30,42,43^

Interestingly, a reduction (of marginal significance) in depressive symptoms of SG subjects was observed after the intervention. This finding may be explained by the creation of new friendship ties and exposure to others facing similar cognitive problems. The opportunity to do the training and improve performance may also have had a positive impact on depressive symptoms.^12,32^

The current study suggests that cognitive training in older adults with MCI may represent a therapeutic option able to prevent or delay cognitive or functional decline.^10^ According to Ramos,^44^ cognitive performance and dependence are modifiable factors associated to risk of death. In sum, cognitive interventions akin to the one applied in this study can contribute toward promoting health and independence among the elderly. Further investigations in larger samples are now warranted to verify the findings of the present study.
